# Quantitative comparison of DNA detection by GFP-lac repressor tagging, fluorescence in situ hybridization and immunostaining

**DOI:** 10.1186/1472-6750-7-92

**Published:** 2007-12-20

**Authors:** Il-Han Kim, Jens Nagel, Simone Otten, Boris Knerr, Roland Eils, Karl Rohr, Steffen Dietzel

**Affiliations:** 1 University of Heidelberg, BIOQUANT, IPMB, and German Cancer Research Center (DKFZ), Dept. Bioinformatics and Functional Genomics, Biomedical Computer Vision Group, Im Neuenheimer Feld 267, 69120 Heidelberg, Germany; 2 Ludwig-Maximilians-Universität München, Department Biologie II, Planegg-Martinsried, Germany; 3 Ludwig-Maximilians-Universität München, Walter-Brendel-Zentrum für Experimentelle Medizin, Marchioninistr. 27, D-81366 München, Germany

## Abstract

**Background:**

GFP-fusion proteins and immunostaining are methods broadly applied to investigate the three-dimensional organization of cells and cell nuclei, the latter often studied in addition by fluorescence in situ hybridization (FISH). Direct comparisons of these detection methods are scarce, however.

**Results:**

We provide a quantitative comparison of all three approaches. We make use of a cell line that contains a transgene array of lac operator repeats which are detected by GFP-lac repressor fusion proteins. Thus we can detect the same structure in individual cells by GFP fluorescence, by antibodies against GFP and by FISH with a probe against the transgene array. Anti-GFP antibody detection was repeated after FISH. Our results show that while all four signals obtained from a transgene array generally showed qualitative and quantitative similarity, they also differed in details.

**Conclusion:**

Each of the tested methods revealed particular strengths and weaknesses, which should be considered when interpreting respective experimental results. Despite the required denaturation step, FISH signals in structurally preserved cells show a surprising similarity to signals generated before denaturation.

## Background

The consistency of fluorescence detection signals with the in vivo distribution of the detected structure is an important technical issue in modern cell biology. Quality of generated signals may be influenced by applied fixation methods [1-3] as well as the approach used for detection. For the detection of specific DNA sequences in the cell nucleus, two methods are available [4]: fluorescence in situ hybridization (FISH) can be applied to any sequence large enough to generate sufficient hybridization sites for DNA-probes, but only to fixed cells. In vivo labeling is possible with GFP fusions to DNA binding proteins such as the lac repressor which then binds to lac operator sequences within transgenes [5]. By design this approach does not label endogenous eukaryotic sequences, except for some tandem repetitive sequences such as centromeres and telomeres [4]. In investigations of the cellular localization of proteins, fusions to fluorescent proteins or immunostaining are commonly applied methods. Despite the widespread usage of these methods, however, simultaneous or sequential application to the same cells are scarce [6] and we are not aware of a detailed comparisons of these detection methods.

Here we provide a qualitative and quantitative comparison of signals from multi-labeling experiments where we applied the labeling methods mentioned above to the same structure. We used a mouse erythroleukemia (MEL) cell line that contained a large array with lac operator transgenes [7], which was in vivo labeled with GFP lac repressor fusion proteins expressed by the cells. Cells were fixed with buffered formaldehyde to maintain structural integrity [3,8], permeabilized, immunostained and three-dimensional image stacks of GFP and immunostaining signals were recorded by fluorescence microscopy. Afterwards, cells were unmounted, subjected to FISH, relocated under the microscope and FISH-signals and again immunostaining-signals were recorded. Signals from all four recorded image stacks were then compared qualitatively and by quantitative digital image analysis. In additional experiments, we tested the similarity of two FISH signals generated by different probes against the same DNA sequence and for differences of GFP and FISH signals when no RNAse digestion was performed.

## Results

### Verification of the image analysis approach with dual color FISH

**Figure 1:**
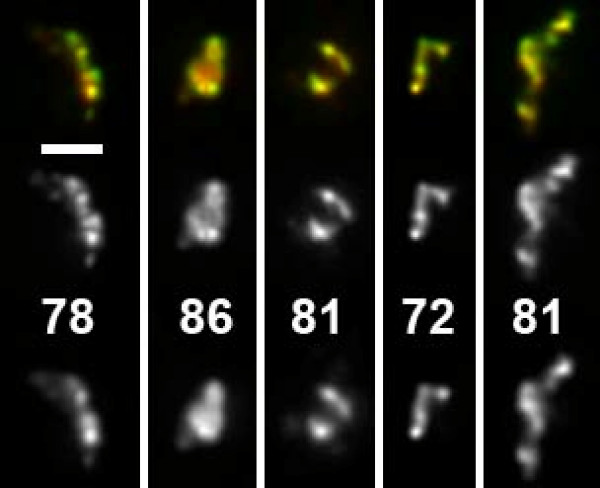
Dual color FISH with two different plasmids detecting the same transgene array. This 50 Mbp transgene array is composed of multiple transgene copies as well as host DNA. pPALZ8.8 (green and center, labeled with FITC) was originally used to generate the transgene array and thus completely covers transgenes while pPS8.8 (red, bottom, labeled with Cy5) detects only the lacO sequence which comprises one sixth of the complete transgene length. CC values are given in percent. They were calculated in 3D, thus they do not necessarily reflect similarity in the projections of deconvolved image stacks shown here. Scale bar: 2 μm.

To quantitatively compare detection signals generated by different techniques, we developed a procedure to calculate a correlation coefficient (CC). The CC is always between +1 (identical signal shape, fully correlated) and -1 (inverse signal shape, anti-correlated). 0 stands for no correlation. To determine which values could be expected under practical conditions for two detection signals from the same structure, we first compared FISH signals generated by two simultaneously hybridized DNA probes labeled in different colors. Both probes were targeted at the transgene array in PALZ39E cells. This array was previously described to have a length of 50 Mbp, containing over thousand copies of the transgene and also intermingling host DNA [7]. One probe contained the whole plasmid used for generation of the transgenic cell line, the other contained only the lac operator repeats and thus one sixth of the total length (see methods). Visual inspection of the signals revealed very similar appearances in both color channels although minor differences could be discerned (Figure 1). Quantitative evaluation of 57 deconvolved image stacks of nuclei revealed CC values between 0.63 and 0.92 with a median value of 0.81 (average 0.80). When the correlation was determined for the same nuclei without prior deconvolution, we obtained a much higher median of 0.94 (average: 0.93, range: 0.79 – 0.97). This was not unexpected, since deconvolution removes blur from the image stacks and blurred images will generally be more similar to each other, even if the underlying signal is not.

Since a labeled substructure in one channel which is not present at exactly the same site in another channel will lead to a decrease of the CC value, we assumed this value to be very sensitive to structural differences of compared signals. We tested this assumption by computationally shifting one of the color channels 0.085 μm in x-direction, 0.065 μm in y-direction and 0.011 μm in z-direction, with subsequent tri-linear interpolation to obtain an image stack with voxels at the same position as the original stacks. Indeed, the median dropped from 0.81 to 0.71 for deconvolved image stacks while the more blurry, non-deconvolved image stacks showed only a drop from 0.94 to 0.91.

### Comparison of multiple detection signals: in vivo GFP-lac repressor staining, antibody detection and FISH on the same transgene array

**Figure 2:**
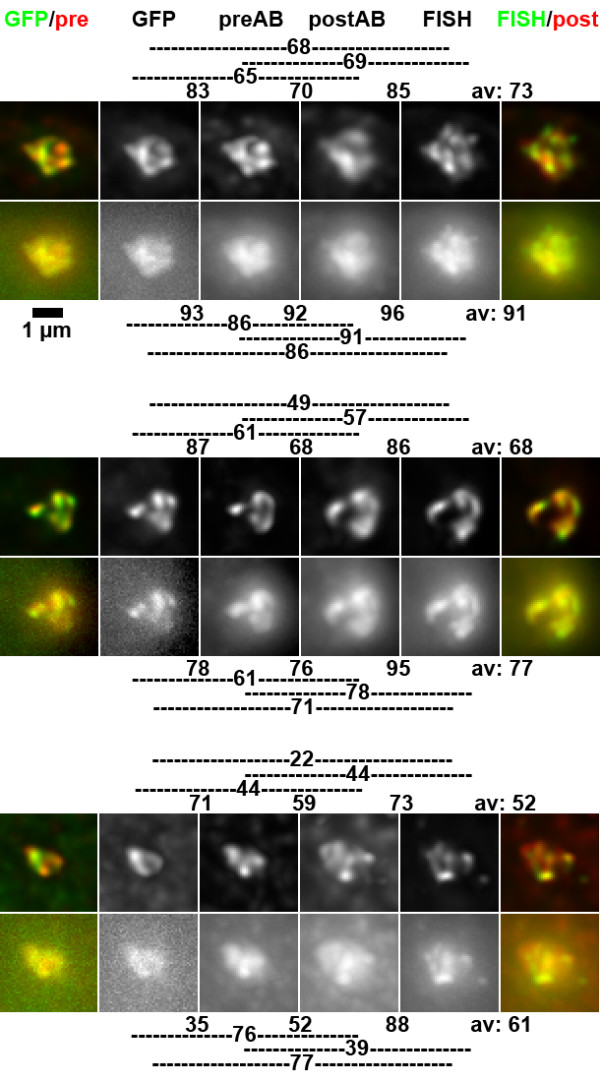
Comparison of GFP, immunostaining and FISH signals from the same nuclear structure. Upper rows show projections of deconvolved image stacks, lower rows those from non-deconvolved images. As indicated at the top of the panel, color overlays on the left are from GFP (green) and antibody signals before FISH (preAB, red, Cy5), color overlays on the right from antibody signals after FISH (postAB, red, Cy5) and FISH signals (green, FITC). Indicated CC values are based on pair-wise comparisons of 3D image stacks, thus they do not necessarily reflect similarity in the projections. Lines on the side of CC values indicate which signals were compared. Shown are examples for high to average CC values (top and center) and low CC values.

In formaldehyde fixed cells, the signal of the GFP-lac repressor bound to lac operators in the transgene array in PALZ39E cells was additionally labeled by immunostaining with anti-GFP antibodies (Figure 2). As expected, qualitative visual inspection showed that GFP and anti-GFP signals were very similar. Detailed examination, however, revealed a number of differences in substructures of the two signals (Figure 2). Deviations occurred both ways, but absence of a GFP signal at a site labeled by antibody detection was more frequent. We assume this to be caused by antibody detection of GFP molecules that were not fluorescent (misfolded or quenched). Unspecific binding of the antibody is unlikely, since these structures were present in the direct neighborhood of the GFP-signal but not remote. For the reverse case, penetration problems of the antibody can be assumed. Immunostaining signals were generally brighter than GFP signals, the latter often obscured by low signal intensity and high nuclear background.

After recording of GFP and antibody signals, a postfixaton, subsequent FISH and again immunostaining was performed (see methods). Visual inspection revealed highly similar appearance of FISH and immunostaining signals, as well as similarity to signals recorded before FISH (Figure 2). Sometimes the FISH signal showed more substructure and higher contrast than other signals (Figure 2, top row). Also, we had the impression that both signals obtained after FISH occupied a larger volume than those obtained before FISH.

Therefore, we first measured volumes after segmenting signals by thresholding. The volume obtained for an individual signal by this approach may vary largely, depending on the subjectively chosen threshold. However, when signals are segmented by steady criteria and volume ratios of series of signals are compared to each other, the obtained ratios are reasonably stable. Because of the difficulties described we limited volume measurements to deconvolved signals with their improved signal to noise ratio. Volumes of GFP signals and simultaneously detected immunostaining were not distinguishable (mean 0.4 μm^3 ^for both, 427 and 460 voxels, respectively). While FISH signals (0.5 μm^3^, 541 voxels) were only 27% larger than GFP signals and 18% larger than pre-FISH antibody signals, antibody signals after FISH (0.7 μm^3^, 794 voxels) showed a 73% volume increase compared to pre-FISH antibody signals. Accordingly, in 53 of 55 nuclei segmented antibody signals were larger after FISH.

**Figure 3:**
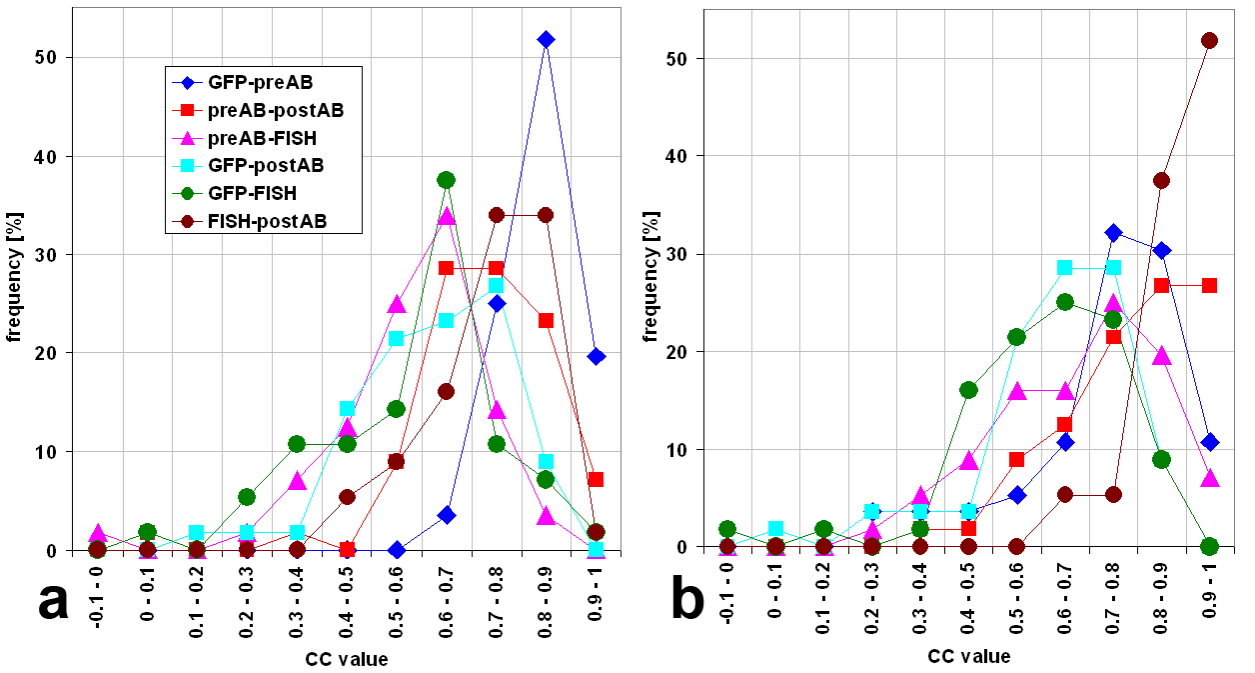
Distribution of values of correlation coefficients. a) deconvolved images. b) non-deconvolved images. Values for 55 processed nuclei were categorized to intervals of 0.1. Values smaller than -0.1 did not occur. While in deconvolved images high CC values were less frequent than in non-deconvolved images, both show the highest CC values for GFP – pre-FISH antibody signal (preAB), FISH – post-FISH antibody signal (postAB) and preAB – postAB.

We next performed a quantitative similarity analysis of signal structures. While shift correction to correct for chromatic aberration was sufficient when simultaneously recorded signals were compared, an image registration procedure was applied to align images recorded before and after FISH (see methods for details). To speed up computational signal comparisons, calculations were performed only within a region of interest which was defined by setting a low threshold to the signals of the four stacks and combining the segmented volumes. While signal to noise ratios in non-deconvolved images were good for immunostaining and FISH signals, for GFP-signals they were too weak to define a threshold for segmentation in 53 of 55 cases. The region of interest was then defined by combining the volume covered by the other three signals. After deconvolution, only 4 of 55 GFP image stacks would not allow thresholding. The correlation coefficient (CC) was calculated for each pair wise comparison of the respective 3D-image stacks, resulting in six CC values per nucleus (Figures 2, 3). For 55 nuclei from four independent experiments, all CC values were calculated (Figure 3, Table 1). High correlations were found between those signals recorded simultaneously (GFP – preAB and FISH – postAB) and between the two antibody signals while the remaining comparisons, including GFP – FISH revealed lower correlations (Figure 3, Table 1). The highest CC value for deconvolved images was found between GFP signals and simultaneously recorded antibody signals with a median CC of 0.86. This value is higher than the 0.81 obtained above for two simultaneously hybridized FISH probes.

**Table 1:** Correlation coefficient values

	Original data						Deconvolved data					
	
	GFP-preAB	preAB-postAB	preAB-FISH	GFP-postAB	GFP-FISH	FISH-postAB	GFP-preAB	preAB-postAB	preAB-FISH	GFP-postAB	GFP-FISH	FISH-postAB
	
Average	0.74	0.78	0.68	0.63	0.62	0.88	0.84	0.72	0.58	0.62	0.58	0.74
Median	0.78	0.83	0.70	0.65	0.65	0.91	0.86	0.76	0.61	0.62	0.62	0.76
Max	0.93	0.97	0.95	0.87	0.86	0.98	0.95	0.94	0.85	0.88	0.90	0.90
Min	0.24	0.38	0.22	0.06	-0.10	0.62	0.62	0.09	-0.05	0.17	0.05	0.45
sdev.	0.16	0.15	0.17	0.16	0.17	0.09	0.07	0.14	0.15	0.15	0.17	0.11
sem	0.02	0.02	0.02	0.02	0.02	0.01	0.01	0.02	0.02	0.02	0.02	0.01

Correlation coefficient (CC) values for pair wise comparisons of different signals from the same structure were calculated for 56 nulcei. preAB is the antibody signal before FISH, postAB the one after FISH. Averages, medians, maximal and minimal values as well as standard deviation (sdev) and the standard error of the mean (sem) are given.

**Figure 4:**
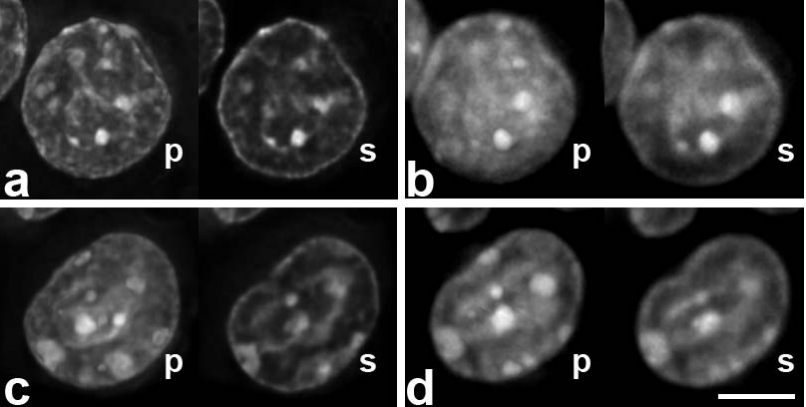
Two counterstained nuclei before (a, c) and after FISH (b, d). Shown are projections (p) and single sections (s) of deconvolved image stacks. Nuclei after FISH are generally more blurry. Single sections from before and after FISH were selected manually to approximate the same nuclear depth. Scale bar 5 μm.

When we compared DAPI-counterstained nuclei before and after hybridization, we noticed that they appeared more blurry after FISH, in agreement with earlier studies [3,8]. The difference was particularly visible in deconvolved image stacks where nuclei appeared quite crisp before FISH (Figure 4). Projections of non-deconvolved nuclei appeared blurry already before FISH (not shown).

### Contribution of DNA-RNA hybridization to FISH signals

**Figure 5:**
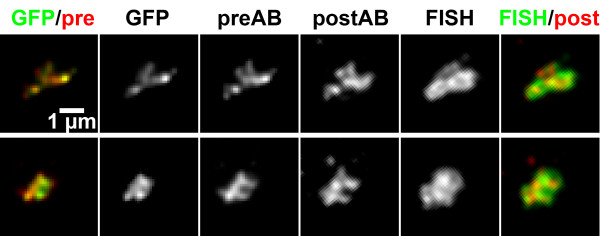
Comparison of GFP, immunostaining and FISH signals without RNAse digestion, allowing for detection of RNA from the transgene arrays by the FISH probe. For panel labeling see Figure 2. Projections of deconvolved images are shown. Note the larger volume of FISH signals compared to other signals. In this experiment, due to the large differences in signal appearance, pre- and post-FISH signals could not be subjected to automated image processing. Therefore, for this experiment only, post-FISH images were matched to the orientation of pre-FISH images manually.

To estimate the potential contribution of DNA-RNA hybridization to FISH signals, we repeated the four signal comparison but now without RNase digestion. The transgene array includes β-galactosidase reporter genes. X-gal staining revealed expression in about 40 percent of cells. DNA of the complete plasmid used for generation of the transgenic cell line was labeled and applied as FISH probe, to allow for potential DNA-RNA hybridization. Visual inspection revealed that compared to GFP or immunostaining signals, FISH signals were often larger, labeling the volume surrounding the other signals (Figure 5). The anti-GFP immunostaining signal after FISH did not match this increase, resembling the anti-GFP signal before FISH. This argues for a contribution of RNA bound probe to the FISH signal in this experiment without RNase digestion. Since our quantitative image analysis approach was designed for very similar signals, we could not reasonably apply it to this data set.

## Discussion

In this study, we quantitatively compared three detection methods widely used in studies of nuclear organization and beyond. Reassuringly, GFP, antibody detection and FISH produced signals similar in overall structure. However, we also could observe differences in details. Anti-GFP antibody signals at sites without GFP fluorescence argue for non-fluorescent GFP molecules, masking a part of the underlying structure. We have not formally proven that such an incomplete detection of GFP fusion proteins by GFP fluorescence also occurs in vivo, before fixation, but this appears as the most likely conclusion. Whether non-fluorescent GFP plays a role in fusion proteins with other partners is unclear at present. On the other hand, antibody detection can be hampered by incomplete penetration. In normal immunofluorescence assays it is difficult to estimate the magnitude of this problem. By using a fluorescent protein as target we could show that the preparation procedure applied here allows for nearly complete detection of fluorescent GFP in this cell type.

FISH signals showed a high similarity with the post-FISH antibody signals. We did not find that FISH signals would have less substructure than GFP signals, as it was described in a previous study [5], maybe as a consequence of harsher denaturation. On the contrary, some FISH signals had more substructure and higher contrast than the other three signals. It is possible that FISH is more sensitive, due to a larger number of fluorochromes per volume and a resulting higher signal to noise ratio, or due to an incomplete binding of the GFP-lac repressor to its target sites. Alternatively, this additional substructure may be caused by moving of the DNA during the denaturation step and the accompanying destruction of the ultrastructure which has been shown by electron microscopy [8]. Post-FISH antibody signals were on average 73% larger than pre-FISH antibody signals, suggesting a certain spreading of the DNA together with DNA bound proteins, during FISH. Surprisingly, FISH signals themselves showed volumes only 27 or 18% larger than GFP- or pre-FISH antibody signals, maybe due to differences in detection efficiency. Changes during FISH, more specifically during the required denaturation step, were described in earlier studies [3,8] and are reflected by the blurrier appearance of DAPI stained nuclei after FISH.

Immunostaining signals showed a high similarity to each other as well as to the simultaneously recorded GFP or FISH signal. Compared to other comparisons, however, the correlation between the two antibody signals from the same nucleus may have been positively influenced by the fact that it was these two signals that were used to align pre- and post-FISH image stacks. Using these signals for alignment of pre and post-FISH stacks seems to be the most reasonable approach, however, since here the same molecules are detected. Despite this favorable situation, in half of the deconvolved nuclei a CC value of only 0.76 or less was reached, further supporting the notion of changes in the underlying structure during denaturation.

## Conclusion

In a previous study, we could show that the appearance of chromosomes or chromosomal regions detected by FISH varies substantially, depending on the fixation protocol applied [3]: While formaldehyde fixed nuclei had relatively compact FISH signals, nuclei subjected to hypotonic swelling, methanol acetic acid fixation, dropping on slides and flattening by air drying (2D-FISH) displayed FISH signals with a dispersed structure, suggesting structural disruption [3]. Our current study confirms that the in vivo organization of chromatin is well represented by FISH signals in formaldehyde fixed cells and thus strengthens the conclusion that this is not the case for the more spread-out signals in 2D-FISH preparations. Our current results show that the spatial distribution of GFP, immunostaining and FISH signals from the same structure in structurally preserved cell nuclei are largely overlapping at the light microscopy level, although they are not identical.

Each of the detection methods tested in this study carries its specific set of advantages and disadvantages. The signal-to-noise ratio of GFP signals was much lower than immunostaining or FISH signals. Also, GFP fusion proteins are apparently not always fluorescent, and this non-fluorescent fraction is not distributed equally. Therefore, GFP in vivo staining should not be regarded as being generally superior compared to other detection methods. In addition, despite the undisputed usefulness of GFP fusion proteins, their expression was linked to changes in the physiological state of living cells such as induction of apoptosis [9], dilated cardiomyopathy in transgenic mice [10], impairment of actin-myosin interactions [11,12], inhibition of polyubiquitination [13], and cytokine induction [14]. Interference with the physiological state of the cell can be excluded for staining techniques applied after fixation. However, antibody detection could be limited by the permeability of the sample, although the permeabilization we applied in the current study allowed comprehensive detection of fluorescent GFP. FISH, by design, requires denaturation of the DNA and thus a partial structural destruction of the sample. Giving this unavoidable disadvantage, we were actually surprised how similar post FISH signals still are on the light microscopic level in structurally preserved cell nuclei when compared to detection prior to denaturation.

## Methods

### Cells and dual color FISH

PALZ39E is a mouse erythroleukemia (MEL) cell line stably transgenic for a GFP lac repressor fusion protein and with multiple integrations of the 15 kbp plasmid pPALZ8.8 containing 64 repeats (2.5 kbp) of the lac operator binding site (lacO), β-globin regulatory sequences and a β-galactosidase reporter gene [7]. The plasmid pPS8.8 [5] also contains 64 copies of the lac operator but no β-globin or β-galactosidase related sequences. pPALZ8.8 and pPS8.8 were labeled by nick translation with Digoxigenin-dUTP or Biotin-dUTP, respectively. Fixation with 3.7% freshly made buffered formaldehyde (10 min), permeabilization treatment and FISH conditions were as described [3] (15 min 0.5% Triton, five freeze/thaw cycles in liquid nitrogen, 10 min 0.1 N HCl, no protease treatment, denaturation in 50% formamide/SSC at 75°C for 2 min). Detection was with Sheep-α-Dig-FITC (1:100, Roche Diagnostics, Mannheim, Germany) and Streptavidin-Cy5 (1:200, Rockland, Gilbertsville, PA).

### Antibody detection of GFP

Cells were cultivated and fixed as above except that grided coverslips (Belco Biotechnology, Etched GRID coverslip 23 × 23 mm, stock No. 1916-92525, distributed by Electron Microscopy Sciences, Ft. Washington, PA, (#72264-23) and obtained through Science Services, Munich, Germany (#141204)) were used to allow relocation of cells. For immunostaining, cells were permeabilized for 10 minutes with 0.5 % Triton X-100 in PBS. Blocking was for 60 minutes or longer with 4% bovine serum albumine in PBS, incubation with primary antibody (RabbitαGFP, 1:500, Invitrogen, R970-01) was for 45 minutes or longer. After 3 washes (ea. 10 min) in PBS, a secondary, biotinylated GoatαRabbit (1:100 Biosource, Camarillo, CA) was applied which was finally detected with Cy5-conjugated streptavidin. Usage of a biotinylated antibody ensured that the signal would be detectable also later, after the denaturation required for FISH. Cy5 is spectrally sufficiently separated from GFP (or FITC) to exclude bleed-through from one fluorescence channel to the other. Nuclei were counterstained with DAPI, mounted with Vectashield (Vector, Burlingame, CA, USA) and fixed with a nail polish that did not disturb GFP fluorescence on microscopic slides for microscopic observation.

### Microscopy

Microcopy was performed on a VisiScope Cell Explorer (Visitron Systems, Puchheim, Germany) based on a Zeiss Axiovert 200 mot microscope and a Spot RT-SE6 CCD Camera with Sony ICX285 chip, controlled by Metamorph Software. 3D-stacks were recorded with a 100 × N.A. 1.4 Zeiss PlanApo oil objective with a voxel size of 0.065 × 0.065 × 0.2 μm except for the multi-signal comparison without RNAse where voxels of 0.103 × 0.103 × 0.25 were obtained with a 63 × NA 1.4 PlanApo objective. The following filter sets were used: DAPI (360/40, 400LP, 470/40), GFP (470/40, 497LP, 522/40), and Cy5 (622/36, 647LP, 667/30). Deconvolution was performed with Huygens essential software (SVI, Hilversum, The Netherlands).

### FISH after recording GFP and immunostaining signals

After the first round of 3D-microscopy, coverslips were unmounted from microscopic slides, washed several times in PBS and subjected to a post-fixation in 1% buffered formaldehyde for 10 minutes to fix the antibodies to their locations. To exclude hybridization of probe to RNA, digestion with DNAse free RNAse A (0,2 mg/ml in PBS for 24 hours at 37°C; Quiagen, Hilden, Germany) was performed except otherwise noted. Control experiments with Acridine Orange staining showed that cytoplasmic and nucleolar RNA was completely removed by this procedure (data not shown). Permeabilization treatment and FISH conditions were as described [3] (see also above). The plasmid pPS8.8 [5] was used as probe, labeled with Digoxigenin-dUTP, except otherwise noted. Control experiments showed that fluorescence from GFP was completely lost after FISH (data not shown). All three steps of the antibody detection of GFP (see above) were repeated. In parallel, Digoxigenin detection was performed with Sheepα Dig-FITC (1:100, Roche Diagnostics). Microscopy and deconvolution were as described above. Comparison of the DNA counterstain appearances before and after FISH ensured correct identification of nuclei.

### Image analysis

Volumes of fluorescent signals were determined with the plug-in Voxel Counter of the freely available open source software ImageJ [15] after threshold segmentation.

To quantify the structural similarity between GFP-, immunostaining- and FISH-signals, we developed a rigid registration approach. The whole procedure was applied to non-deconvolved as well as deconvolved data. Each 3D multi-channel image stack was corrected for chromatic aberration (measured with polychromatic beads) and cropped to the same image dimensions. For faster computational processing, calculations were performed only within a region of interest, defined by setting a low threshold to the signals of all four stacks and combining the segmented volumes. Since here it was more important not to miss any signal parts rather than to accurately reflect signal borders, as was attempted for volume measurements described above, these thresholds were newly determined for each image. A subsequently applied connected-components labeling algorithm identified the largest region in each GFP- or Cy5-channel, which was invariably the signal of interest, as confirmed by visual inspection.

The two multi-channel image stacks of the same nucleus, acquired before and after FISH, were aligned in the 3D-image space by an automatic multi-step registration procedure. This ensured that differences solely due to different alignment during microscopic recording did not influence the measurement results. First, a coarse rigid registration was performed. Since the signal to noise ratio of GFP signals was often very low, the registration was based on the center-of-gravity of the segmented regions of the immunostaining (Cy5-) signals. Second, to refine the rigid registration, a quaternion-based rigid registration scheme was used, which minimizes the mean-squared intensity error by a gradient-descent optimizer. Using the image stack recorded before FISH as reference, the calculated registration transformation, consisting of 3D translation and rotation, was applied to the multi-channel image stack obtained after FISH. Finally, the similarity between all combinations of the GFP-immunostaining and FISH signals was measured. To this end, the overlapping volume of the combined pre- and post-FISH immunostaining signals as well as the FISH signal was determined. GFP signals were disregarded in the creation of this volume because of their low signal to noise ratio. The coefficient for the normalized cross correlation (CC), however, was calculated pair wise for all four signals according to the formula

$$CC=\frac{\sum \left(f(x,y,z)-m_{f}\right)\left(g(x,y,z)-m_{g}\right)}{\sqrt{\sum {\left(f(x,y,z)-m_{f}\right)}^{2}}\sqrt{\sum {\left(g(x,y,z)-m_{g}\right)}^{2}}}$$

where *f *and *g *denote the two signals and *m*_*f *_and *m*_*g *_the corresponding mean values.

## Authors' contributions

Wet lab experiments and microscopy were performed by SO and BK, image processing and volume measurements by JN. I-HK performed image registration and correlation analysis, supervised by KR and RE. SD conceived the study, supervised SO, BK and JN and wrote the manuscript.
